# Evaluation of the Application Effects of *Siniperca chuatsi* in Biofloc Systems: A Comparative Study on the Use of Bamboo Flour and Rice Straw as Carbon Sources

**DOI:** 10.3390/microorganisms13071631

**Published:** 2025-07-10

**Authors:** Huiling Zhang, Zhaojie Deng, Shijun Chen, Xi Xiong, Wenhui Zeng, Fang Chen, Huanjiao Tan, Xuran Chen, Canmin Yang, Yuhui He, Dizhi Xie, Lian Gan

**Affiliations:** 1 College of Marine Sciences, South China Agricultural University, Guangzhou 510642, China; 2 Nansha-South China Agricultural University Fishery Research Institute, Guangzhou 511457, China; 3 Guangdong Laboratory for Linnan Modern Agriculture, Guangzhou 510642, China

**Keywords:** *Siniperca chuatsi*, rice straw, bamboo flour, water quality, bacterial community

## Abstract

A 56-day trial was conducted to assess the effects of rice straw (RS) and bamboo flour (BF) on growth performance, water quality, gill histology, and the bacterial community of water and the intestine of mandarin fish (*Siniperca chuatsi*) in biofloc technology systems. The results showed that mandarin fish in the RS and BF groups had comparable survival rates of 100.00 ± 0.00 and 93.33 ± 3.85%; feed conversion ratios of 1.13 ± 0.02 and 1.40 ± 0.15; and weight gain rates of 112.21 ± 1.56 and 100.92 ± 6.45%, respectively. From days 11 to 56 of the farming period, the BF group was more effective than the RS group in removing total ammonia nitrogen (TAN) and NO_2_^−^-N, maintaining TAN levels below 0.24 ± 0.05 mg/L. During the early stage of the experiment, the TAN level in the RS group was higher; however, with the supplementation of a carbon source, it gradually decreased and eventually stabilized at 0.13 ± 0.03 mg/L later in the farming period. The secondary gill lamella in the RS group was curved and showed hyperplasia, and the basal gill lamellae showed an increase in the volume of interlamellar cell mass in the BF group. Genes related to denitrification (narG, napA, nirS, nirK, and nosZ) and anammox showed higher expression levels in the BF group than in the RS group, although the differences were not statistically significant (*p* > 0.05). The results of 16S rRNA sequencing research showed that both treatment groups’ intestinal and water bacterial communities had comparable levels of richness and diversity. *Pseudomonas mosselii* was the dominant bacterial species in the water. In the BF group, the dominant intestinal species were *Bacillus halodurans* and *Caldalkalibacillus thermarum*, while in the RS group, the dominant species was *Plesiomonas shigelloides*. In conclusion, rice straw and bamboo flour are applicable in BFT systems for mandarin fish culture, with good growth performance and water quality. The BF group showed higher nitrogen removal efficiency and denitrification gene expression than the RS group.

## 1. Introduction

The mandarin fish (*Siniperca chuatsi*) is a prized aquaculture species in China, with production reaching 477,592 tons in 2023 [1]. Due to its fast growth, high market value, and superior meat quality, it is extensively farmed across China. However, because mandarin fish are a predatory species, their peculiar feeding habits have traditionally limited the advancement of intensive farming practices [2]. The fish typically rely solely on forage fish as their food source, which can increase the risk of disease transmission through pathogens or parasites carried by the forage fish [3,4]. Following years of taming and selective breeding, mandarin fish are now able to sustainably consume formulated diets [5]. Being a carnivorous species, the mandarin fish has a high dietary protein requirement, which is as much as 44.78% and can be increased up to 62.2% in commercial diets [6,7], which leads to an increase in nitrogenous emissions, such as total ammonia nitrogen (TAN) and nitrite nitrogen (NO_2_^−^-N) in the pond [8]. Consequently, these emissions pose a threat to aquatic species, including fish, due to the risks of eutrophication and toxicity [9,10,11]. Specifically, exposure to TAN and NO_2_^−^-N pollution can induce tissue damage, adversely affecting blood oxygen carrying capacity, osmoregulation, and the immune system, ultimately resulting in the death of aquatic species [12,13,14].

Biofloc technology (BFT) is a closed-type method of purifying water based on the C/N ratio of 15, which uses nitrifying bacteria and heterotrophs as well as other related microorganisms to eliminate harmful inorganic nitrogen [15,16,17]. This technology not only boosts the growth and immunity of farmed animals by fostering the growth of beneficial bacteria, but it also eradicates harmful bacteria [18]. As the microbial floc matures, it utilizes the added carbon source as a source of energy and is converted into microbial proteins by absorbing nitrogenous substances in aquaculture water, thereby stimulating bacterial proliferation [19]. The type of organic carbon source somewhat determines the content and structure of microorganisms, thereby influencing the quality of bioflocs [20,21]. Carbon sources for BFT can be classified into simple carbohydrates and complex carbohydrates. These encompass a variety of sources such as molasses [22], glucose [23], cassava starch [24], cornmeal [25], wheat flour [26], rice bran [27], and glycerol [28]. However, these carbon sources require frequent control additions, typically several times per day or every few days, leading to increased production costs [29,30]. Natural materials rich in cellulose, including agricultural wastes and woody materials, have emerged as a cost-effective alternative that eliminates the need for frequent additions [31]. In addition, they can act as a carrier in BFT aquaculture systems to provide sufficient space for nitrifying bacteria and others to attach and grow [30]. Rice straw and bamboo flour are types of agricultural waste that have a rich organic carbon content and are cheap, renewable, and abundant [32,33,34]. BFT that utilizes rice straw as a substrate has been documented and successfully implemented in shrimp aquaculture [35]. On the other hand, nothing is known about using bamboo meal as the only carbon source in BFT aquaculture systems for the culture of shrimp and fish. Therefore, this study aimed to explore the impact of rice straw and bamboo flour on the bacterial population in the water and intestine of mandarin fish in BFT aquaculture systems, along with their growth, water quality, and gill histology.

## 2. Materials and Methods

### 2.1. Start-Up of BFT Aquaculture Systems

The experiment was conducted in 6 fiberglass-reinforced plastic circular tanks (300 L) at the aquatic animal breeding laboratory of South China Agricultural University. The carbon sources utilized in the two treatment groups were BF (bamboo flour) and RS (rice straw). Each treatment had three replicates, and fish were assigned randomly. Rice straw (total carbon content: 42.3%, 40-mesh) and bamboo flour (total carbon content: 49.9%, 40-mesh) were purchased from Lianyungang City, Jiangsu Province, China. Rice straw, bamboo flour, NH_4_Cl, and NaNO_2_ were added based on a C/N ratio of 15, with initial TAN and NO_2_^−^-N levels at 5.36 ± 0.17 and 5.00 ± 0.10 mg/L, respectively. In addition, total alkalinity was adjusted using NaHCO_3_ and maintained at 90–120 mg/L. The BFT aquaculture systems utilized in this experiment were designed as zero-water exchange systems. Water was only added to the tanks to compensate for evaporation and to remove water for quality sampling; all tanks were originally supplied with 250 L of dechlorinated tap water. Air was continuously diffused from the bottom of the six tanks using an air disc by a 60-W blower that was attached to each tank. In addition to supplying sufficient oxygen for fish in culture, this procedure preserved the suspension of organic particles and biomass. Further experiments were started when the TAN and NO_2_^−^-N levels stabilized below 0.05 mg/L [36].

### 2.2. Fish Stocking and Management

Mandarin fish (initial weight: 38.52 ± 0.17 g) was obtained from Xintun Biological Technology Co., Ltd. (Guangdong, China). Ninety mandarin fish were assigned to two treatment groups and randomly distributed into six tanks. The fish were provided with a commercial diet until they appeared to be satiated, twice daily at 8:30 and 16:00. The quantity of feed consumed was measured on a daily basis. The feed had the following composition: crude protein (≥46%), crude fat (≥5%), crude fiber (≤6%), crude ash (≤24%), lysine (≥2.5%), and moisture content (≤12%).

During the trial, the groups were supplied with RS and BF at 20% of the mandarin’s daily feed intake on days 20 and 35, respectively. Water quality parameters were measured: temperature 24.83 ± 0.34 °C, pH 7.36 ± 0.04, Dissolved Oxygen (DO) 7.4 ± 0.11 mg/L, total alkalinity 99.39 ± 2.08 mg/L. The experiment had a duration of 56 days. Before the experiment, the initial body weight (IBW) was recorded. After the experiment, the final body weight (FBW), survival rate (SR), feed intake (FI), weight gain (WG), feed conversion ratio (FCR), and specific growth rate (SGR) were measured. The specific calculation formulas are as follows: SR (%)= 100 × (final number of fish)/(initial number of fish); WG (%) = 100 × (final body weight − initial body weight)/initial body weight; FI (% day^−1^) = total feed intake (g, dry weight) × 100/$\sqrt{\left(\mathrm{IBW}\;\times \;\mathrm{initial}\;\mathrm{number}\;\mathrm{of}\;\mathrm{fish})\;\times \;(\mathrm{FBW}\;\times \;\mathrm{final}\;\mathrm{number}\;\mathrm{of}\;\mathrm{fish}+\mathrm{dead}\;\mathrm{fish}\;\mathrm{body}\;\mathrm{weight}\right)}.$ FCR = feed consumed (g, dry weight)/weight gain (g, wet weight). SGR (% day^−1^) = 100 × [Ln (FBW) − Ln (IBW)]/days.

### 2.3. Sample Collection

Making use of a 0.22 µm filter membrane, 100 mL of water was collected from each tank at the end of the experiment. The South China Agricultural University’s handling requirements were followed during every treatment involving fish. The intestinal contents and gills of three randomly selected sedated fish from each tank were carefully extracted using sterile tools and transferred into sterile cryotubes. Water and intestine samples were refrigerated at −80 °C for further analysis, while gill samples were preserved in 4% paraformaldehyde.

### 2.4. Determination of TAN and NO_2_^−^-N

During the experiment, the levels of TAN and NO_2_^−^-N in the water were assessed every five days with the aid of a water quality analyzer (HDD-05, Dinghai Technology Co. Ltd., Guangzhou, China).

### 2.5. Histopathological Procedure

Following the methods outlined by Chen [37], gill tissues from three fish from each replicate were preserved in 4% paraformaldehyde. The samples were subsequently forwarded to Sevier Biotechnology Co., Ltd. (Wuhan, China) for processing. Hematoxylin and eosin (H&E) staining was followed by photographing the sections using a microscope (Nikon E200MV, Nikon Corporation, Tokyo, Japan).

### 2.6. Bioinformatics Analysis

Majorbio Bio-Pharm Technology Co. Ltd. (Shanghai, China) provided bioinformatics analysis services for this study, as previously described [38]. The effective tags were combined and grouped into 97% sequence similarity operational taxonomic units (OTUs) using the UPARSE process. Data analysis was conducted online using the Majorbio Cloud Platform (https://www.majorbio.com, accessed on 5 May 2025).

### 2.7. qPCR Analysis

The expression of the anaerobic ammonium oxidation (anammox) and denitrification genes was analyzed using quantitative polymerase chain reaction (qPCR). Utilizing the E.Z.N.A.^®^ soil DNA Kit (Omega Bio-tek, Norcross, GA, USA), total DNA was harvested from water microbial samples, followed by an integrity assessment on a 1% agarose gel. Its concentration was then measured using a microvolume spectrophotometer (Thermo Scientific NanoDrop 2000, Thermo Fisher Scientific Inc, Waltham, MA, USA), and water treated with DEPC was used to bring it to a constant concentration at the end. The DNA was amplified for the target gene fragments using the TB Green^®^ Premix Ex TagTM II reagent (TaKaRa Biomedical Technology Co., Ltd., Dalian, China). The target gene fragments were amplified using the TB Green^®^ Premix Ex TagTM II reagent (TaKaRa, China). The 16S rDNA gene served as an internal reference gene for the detection of the target genes (anammox, narG, napA, nirS, nirK, and nosZ) in the water. The primers used in this study were produced by Shanghai Sangon Biotechnology Co., Ltd. (Shanghai, China). Detailed information about these primers can be found in Table S1, and the procedure for their use is outlined in the study by Yan [39]. The 2^−∆∆Ct^ method was used to measure the expression of genes.

### 2.8. Statistical Analysis

The results were presented as the mean ± SEM (standard error of the mean) and examined through an independent samples *t*-test to discover notable discrepancies, with the application of SPSS Statistics 25.0 software from IBM (International Business Machines Corporation, Armonk, NY, USA). A statistically significant result was shown by a probability value of *p* < 0.05.

## 3. Results

### 3.1. Growth Performance

As shown in Table 1, there was no significant difference in the growth performance of mandarin fish between the two treatment groups. After a period of 56 days, both treatment groups exhibited a survival rate exceeding 93% for the mandarin fish. Statistical analysis revealed that there was no significant difference in terms of FBW, WG, FCR, FI, and SGR between the two groups (*p* > 0.05).

### 3.2. Changes of TAN and NO_2_^−^-N in the Water

**Figure 1:**
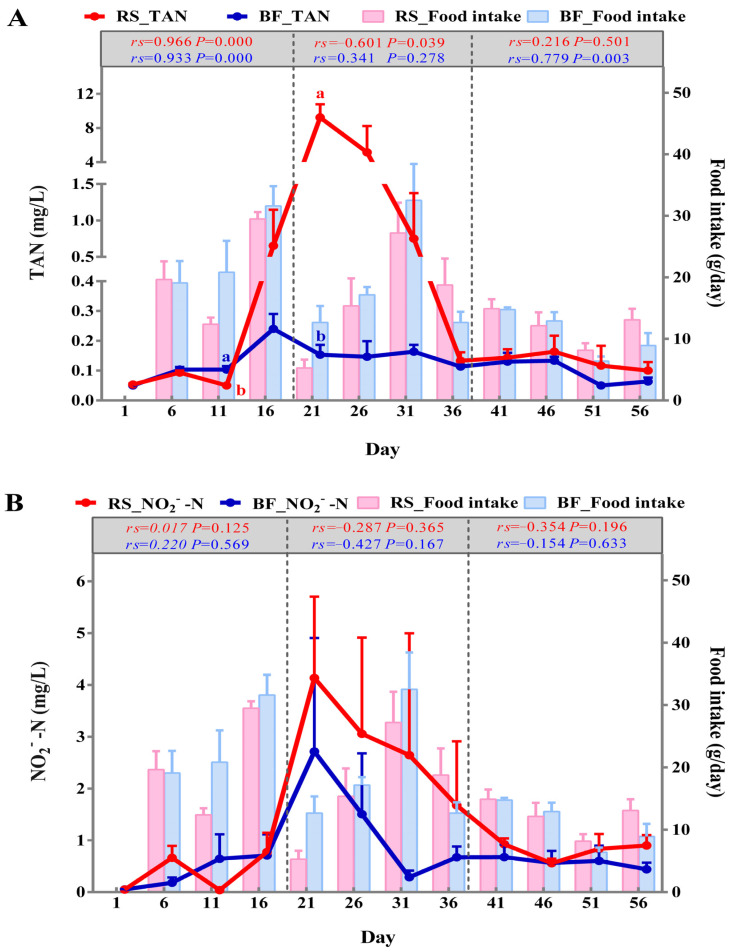
Dynamic changes of TAN (**A**) and NO_2_^−^-N (**B**) in the water and daily feed intake of mandarin fish in the RS and BF groups. Results are presented as the means ± SEM (*n* = 3). Different lowercase letters indicate significant differences between treatments on the same day (*p* < 0.05).

Figure 1A,B depict the changes in TAN and NO_2_^−^-N concentrations, respectively, across the 56-day farming period for both groups. The TAN in the BF group remained below 0.24 ± 0.05 mg/L during the breeding process. However, the TAN in the RS group increased to 9.23 ± 1.55 mg/L from day 0 to day 21, then decreased and stabilized at 0.13 ± 0.03 mg/L on day 36. The trend of NO_2_^−^-N levels in the RS group was similar to that in the BF group, with no significant difference observed (*p* > 0.05). The NO_2_^−^-N in the RS and BF groups increased from day 0 to day 21, and their concentrations peaked at 4.13 ± 1.57 and 2.71 ± 2.19 mg/L, respectively, and then decreased. The NO_2_^−^-N in the RS group decreased to 0.92 ± 0.11 mg/L on day 41, and the BF group decreased to 0.29 ± 0.13 mg/L on day 31, and then both groups stabilized.

Due to the elevation of TAN and NO_2_^−^-N from 21 to 36 days, the water quality dynamics of the 56-day period were divided into three phases: water quality stabilization, water quality fluctuation, and water quality recovery. Spearman correlation analysis was carried out between daily feed intake and water quality parameters, and the results of the analysis are shown in Figure 1A,B. In the BF group, daily feed intake showed an extremely significant positive correlation with TAN (*p* < 0.01), except for days 21–36. A statistically significant negative connection (*p* < 0.05) was observed between daily feed intake and TAN in the RS group, specifically during the period of days 21–36 when TAN levels were high. However, no correlation was found between daily feed intake and NO_2_^−^-N (*p* > 0.05) in either of the two treatment groups during the farming period.

### 3.3. Gill Histology

**Figure 2:**
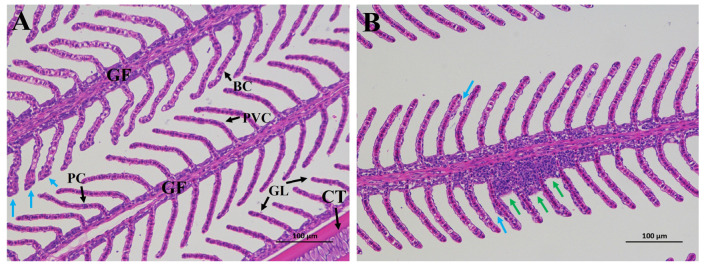
Histological observations were made on the gills of mandarin fish after 56 days of breeding in bamboo flour and rice straw substrate-based BFT aquaculture systems, The RS group is shown in (**A**), and the BF group is shown in (**B**). GF, gill filament; GL, secondary gill lamella; CT: Chondrocyte; PVC, pavement cell; PC, pillar cell; BC, blood cell; the volume of interlamellar cell mass increased (The green arrow in **B**); the secondary gill lamella with hyperplasia (The blue arrows in **A** and **B**). H&E staining, magnification 200×, scale bar = 100 µm.

As shown in Figure 2, the gill tissues of mandarin fish were structurally intact in both groups but showed different degrees of tissue damage. In the RS group, some of the secondary gill lamella on both sides of the gill filaments were curved, and some of the secondary gill lamella showed hyperplasia (blue arrows). Compared with the RS group, the secondary gill lamella shows slight hyperplasia in the BF group, while the basal gill lamellae show an increase in the volume of interlamellar cell mass (green arrows).

### 3.4. Microbiota Diversity Analysis

This study yielded a total of 560,417 valid bacterial sequences via Illumina sequencing analysis. After standardizing all samples, the bacterial OTUs amounted to 6363. Between water and intestinal samples, similar OTUs and alpha diversity indices were found for both groups (*p* > 0.05) (Table 2). The BF group exhibited higher OTUs and richness than the RS group in both intestinal and water samples; however, these differences were numerical rather than statistically significant.

**Figure 3:**
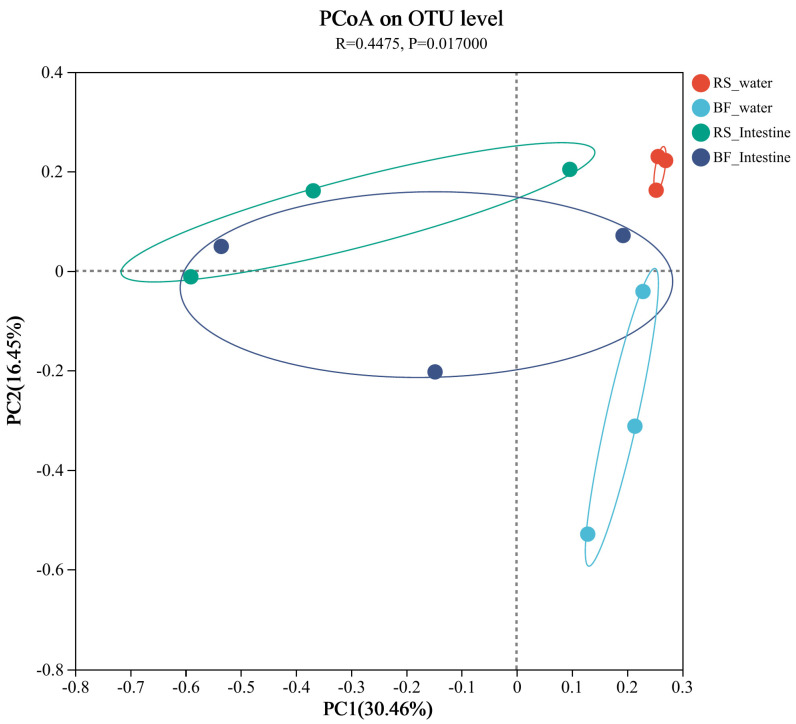
The PCoA analysis of β-diversity, which includes the water and intestine of mandarin fish. Results are presented as the means ± SEM (*n* = 3).

As shown in Figure 3, the investigation analyzed the microbial composition of the water and intestines of mandarin fish belonging to the treatment groups. Mandarin fish showed a separate but strongly overlapping intestinal bacterial community in both treatment groups (ANOSIM, R = 0.4475, *p* = 0.017).

### 3.5. Bacterial Community Composition

**Figure 4:**
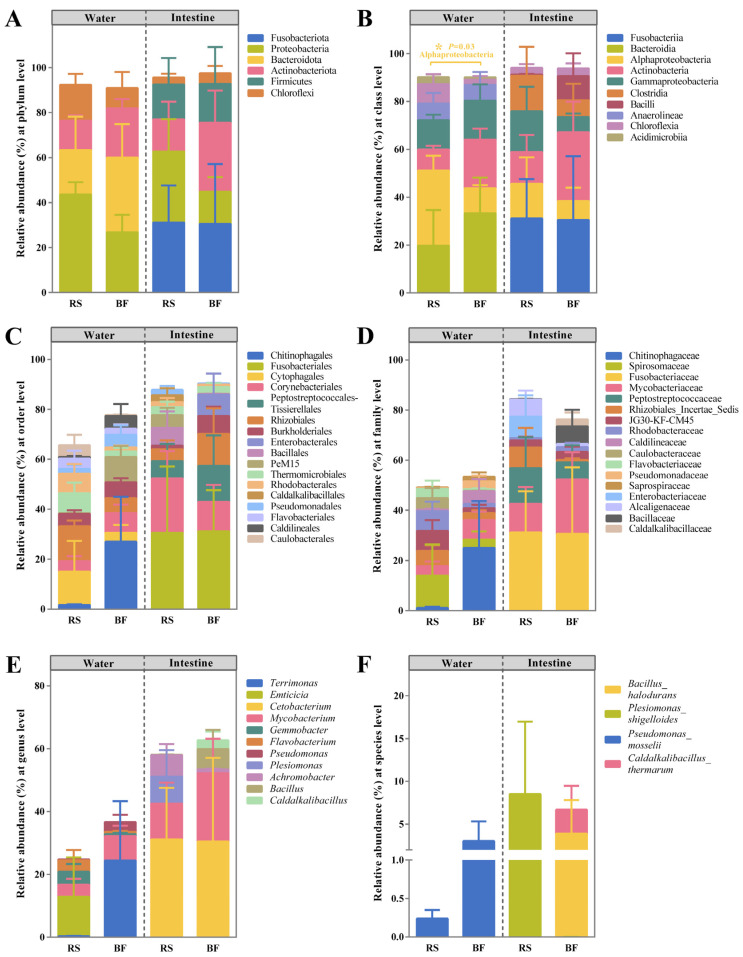
Relative abundance of bacteria at phylum (**A**), class (**B**), order (**C**), family (**D**), genus (**E**), and species (**F**) levels (more abundant than 5%, except for unclassified). Results are presented as the means ± SEM (*n* = 3). “*” means significant difference (*p* < 0.05).

The relative prevalence of bacteria in the RS and BF groups was analyzed by examining the bacterial communities in the water and intestines from both treatments at several taxonomic levels (Figure 4).

The bacterial groups that dominated the water samples from both treatments, classified by phylum, were Proteobacteria, Bacteroidota, and Actinobacteriota. However, the RS group had a higher proportion of Proteobacteria (RS group, 43.58%; BF group, 26.74%), while the BF group had a higher proportion of Bacteroidota (RS group, 19.78%; BF group, 33.30%) and Actinobacteriota (RS group, 13.05%; BF group, 21.78%) in the water samples. Contrarily, Fusobacteriota, Proteobacteria, and Actinobacteriota emerged as the dominant bacterial groups in the intestinal samples from both treatments. The RS group had a higher proportion of Proteobacteria (RS group, 31.13%; BF group, 30.49%), while the BF group had a higher proportion of Actinobacteriota (RS group, 14.31%; BF group, 30.72%).

At the class level, the primary bacterial communities in the water for both treatments were Bacteroidia (19.77%; 33.30%), Alphaproteobacteria (31.36%; 10.49%), Actinobacteria (8.88%; 20.26%), and Gammaproteobacteria (12.19%; 16.25%). Interestingly, the RS group demonstrated a significantly elevated relative abundance of Alphaproteobacteria in comparison to the BF group (*p* < 0.05). In the intestine, the primary bacterial communities for both treatments were Fusobacteriia (31.13%; 30.49%), Actinobacteria (13.24%; 28.71%), Gammaproteobacteria (17.08%; 6.37%), and Alphaproteobacteria (14.49%; 7.89%).

At the order level, Chitinophagales and PeM15 were more prevalent in the water of the BF group than in that of the RS group, whereas Cytophagales and Rhizobiales were less abundant. Contrarily, the intestinal samples from the BF group exhibited a higher abundance of Peptostreptococcales-Tissierellales and Rhizobiales compared to the RS group, while the abundance of Corynebacteriales was lower.

Within the family level, the RS group’s water samples exhibited a higher abundance of Spirosomaceae and JG30-KF-CM45 compared to the BF group, whereas Chitinophagaceae and Mycobacteriaceae were found in lower quantities. Contrarily, the intestinal samples from the RS group exhibited a higher abundance of Peptostreptococcaceae and Rhizobiales_Incertae_Sedis compared to the BF group, while the abundance of Mycobacteriaceae was lower.

When compared to the RS group, *Terrimonas* and *Mycobacterium* were more prevalent at the genus level in the water of the BF group, while *Emticicia* was absent. In contrast, *Mycobacterium* and *Cetobacterium* were more prevalent at the genus level in the intestine of the BF group. Additionally, *Bacillus* and *Caldalkalibacillus* were absent from the RS group, but *Plesiomonas* was found.

At the species level, bacteria with a relative abundance greater than 5% in water from both treatment groups are *Pseudomonas mosselii*, which was detected to be more prevalent in the BF group than the RS group. The intestinal samples of both treatments had different dominant species (more abundant than 5%): *Plesiomonas shigelloides* for the RS group and *Bacillus halodurans* and *Caldalkalibacillus thermarum* for the BF group.

### 3.6. Relative Expression Levels of Genes

**Figure 5:**
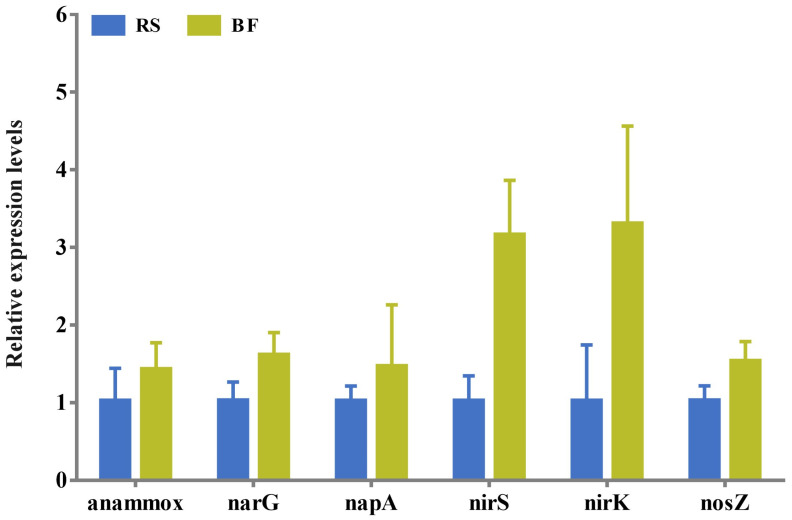
Relative expression levels of anaerobic ammonium oxidation and denitrification genes in water of bamboo flour and rice straw substrate-based BFT aquaculture systems. Results are presented as the mean ± SD (*n * =  3).

As shown in Figure 5, genes related to denitrification (narG, napA, nirS, nirK, and nosZ) and anaerobic ammonium oxidation (anammox) were expressed at higher levels in the water samples from the BF group than in the RS group; however, these differences were not statistically significant *(p* > 0.05). Notably, the denitrification-related genes nirS and nirK were expressed 2.14 and 2.28 times higher, respectively, in the BF group compared to the RS group; this was a numerical difference without statistical significance (*p* > 0.05).

### 3.7. Correlation Analysis for Environmental Factors

**Figure 6:**
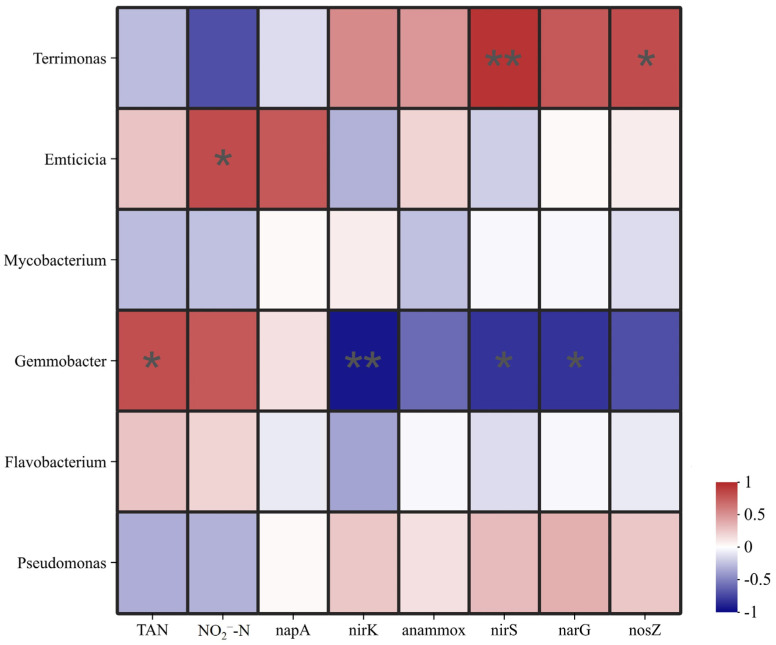
Correlation analysis for the bacterial genus in water and environmental factors (TAN, NO_2_^−^-N, anammox, narG, napA, nirS, nirK, and nosZ) in the habitat of mandarin fish. The color of each cell shows the Spearman correlation coefficient between the corresponding rows and columns. The “*” in the color of each cell represents the significance between rows and columns, “*” means significant difference (*p* ≤ 0.05), “**” means very significant difference (*p* ≤ 0.01).

We analyzed the association of the bacterial community with the aquatic environment of mandarin fish using the Spearman correlation heatmap (Figure 6). The results indicated a positive association between *Gemmobacter* and TAN and a significant inverse relationship with nirK, nirS, and narG (*p* < 0.05). *Emticicia* and NO_2_^−^-N were found to have a significant positive connection (*p* < 0.05). Additionally, *Terrimonas* displayed a significant positive association with nirS and nosZ (*p* < 0.05).

## 4. Discussion

At present, large-scale breeding of mandarin fish in flow-through water tanks and recirculating water systems using formula feeds has been widely reported. The results of these studies show that after 56 days of aquaculture experiments in flow-through water tanks (initial weight: 151 ± 0.55 g, dietary protein: 55.19% DM, temperature: 23.00 ± 0.58 °C) and recirculating water systems (initial weight: 40.70  ±  0.20 g, dietary protein: 47.20% DM, temperature: 21.00 ± 0.50 °C), the mandarin fish WG was 61.92% and 87.26%, respectively, with FCR of 1.83 and 1.71 [40,41]. In this study, the FCR of mandarin fish (initial weight: 38.52 ± 0.17 g, dietary protein: ≥ 46%, temperature: 24.83 ± 0.34 °C) ranged from 1.13 to 1.40, which was lower than that of the flow-through water tanks and recirculating aquaculture systems, and the WG of mandarin fish in the BFT aquaculture systems ranged from 100.92 to 111.21%, which was higher than those previously reported [40,41]. These results highlighted the good growth performance of the fish and shrimp in the BFT aquaculture systems, a result consistent with findings from studies on *Oreochromis niloticus* [42], *Litopenaeus vannamei* [43], and *Cyprinus carpio* [44]. The use of different carbon sources in the BFT aquaculture systems has different effects on the growth performance of aquatic animals [45,46]. At present, species like *Oreochromis niloticus*, *Cyprinus carpio*, *Carassius auratus gibelio*, *Litopenaeus vannamei*, and other organisms have been cultured with the addition of simple carbohydrates (e.g., molasses, glucose, and sucrose), which are commonly used in BFT aquaculture systems, and have exhibited enhanced growth performance [47,48,49,50]. However, these carbon sources require frequent control additions, typically several times per day or every few days, leading to increased production costs [29,30]. This presents a challenge in terms of cost-effectiveness and sustainability. In BFT aquaculture systems, the use of complex carbohydrates not only minimizes the need for frequent operations but also serves as a substrate for microbial attachment and growth [30,31]. The incorporation of complex carbohydrates (like rice bran, starch, and corn) in BFT aquaculture systems has been shown to enhance the growth performance of various aquatic species [44,51]. *Cyprinus carpio* growth performance in BFT aquaculture systems with rice bran as a carbon source outperformed that of sucrose and sucrose + rice bran groups [44]. The importance of complex carbohydrates in BFT aquaculture systems is further supported by the study’s findings. In this study, two types of agricultural waste products, rice straw and bamboo flour, were used as the externally added carbon sources in the BFT aquaculture systems, which have been mentioned in only a few reports. These reports involve species like *Litopenaeus vannamei*, *Tor khudree Sykes*, *Cyprinus carpio*, and *Labeo calbasu*, and their production has increased [35,52,53,54,55]. This further indicates that fish and shrimp culture can be successfully conducted using BFT aquaculture systems, regardless of whether rice straw or bamboo flour is used as the carbon source.

Although the mandarin fish development performance was comparable in the RS and BF groups, variations were noted in the water quality indices during the 56-day farming period. Specifically, starting from day 16 of the farming period, the BF group showed greater efficiency than the RS group in removing TAN and NO_2_^−^-N, while maintaining TAN concentrations below 0.24 ± 0.05 mg/L.This finding aligns with similar results that were obtained with five major species of carp that were cultivated in India utilizing two layers of periphytic substrate (bamboo mats) and demonstrated a strong ability to eliminate TAN and NO_2_^−^-N [54,56]. The reduction in TAN and NO_2_^−^-N in the water is associated with the enhancement of the N-cycle metabolic pathway [57]. The expression level of key enzyme genes in the N-cycle pathway was increased in the BF group, which is key to maintaining the stability of the water quality in the BF group. However, the TAN level of the RS group increased in the early stage of mandarin fish culture and peaked, after which this level gradually decreased and stabilized at 0.13 ± 0.03 mg/L later in the culture period. The accumulation of TAN concentration could be a result of the nitrification process being established in the initial stages of cultivation [44]. This trend is the same as the pattern of water quality changes for mandarin fish in ponds and *Cyprinus carpio* L. in BFT aquaculture systems [47,58,59]. Similarly, this trend was also observed in *Litopenaeus vannamei* culture using straw substrate biofloc and maintaining TAN and NO_2_^−^-N levels at under 1 mg/L throughout the 30 days of the farming period [52]. In addition, the immobilization of heterotrophic bacteria and the growth encouragement of these bacteria by carbon sources are responsible for the decrease in TAN in the RS group [17,45]. It is important to highlight that the elevated levels of TAN and NO_2_^−^-N in the water restrict the daily feed intake of mandarin fish. This observation is consistent with previous studies that have identified a correlation between the concentrations of nitrite and ammonia in aquaculture water and the feeding intake of fish [60,61]. This phenomenon might be due to fish defending themselves against environmental stresses by reducing feed intake and expending more energy [62]. In addition, nitrogen-containing substances in the water often accumulate due to feed inputs, potentially endangering the health of the cultured organisms during the course of aquaculture. In previous reports, the safe levels of TAN and NO_2_^−^-N in mandarin fish culture were 4.865 mg/L (equal to 0.047 mg/L NH_3_) and 7.54 mg/L, respectively [63,64]. However, seven days of high TAN levels (5.14–9.23 mg/L) in the RS group reduced feed intake in mandarin fish. The study findings indicate that high ammonia exposure suppresses appetite, which is in line with earlier findings in rainbow trout [65] and *Rachycentron canadum* [66]. It is well known that the toxicity of non-ionized ammonia (NH_3_) to fish is immediate, and ionized ammonia (NH_4_) is not considered to be harmful [67]. In this study, despite the high concentration of TAN, there were no observed fatalities among the mandarin fish. This outcome could be attributed to the stable pH levels (7.36 ± 0.04) and DO levels (7.4 ± 0.11 mg/L), which contributed to a reduction in the proportion of toxic NH_3_ within the TAN [67,68,69]. On the other hand, it may be due to the BFT aquaculture systems. Studies have indicated that when the TAN concentration in aquaculture water reaches 12.19 mg/L, the immune response and survival rate of *Ompok bimaculatus* are enhanced by the BFT group [70]. This suggests that BFT effectively removes TAN and NO_2_^−^-N from the water and limits toxic NH_3_ proportions within TAN, thereby mitigating its harmful effects on fish.

The gills, which are the main respiratory organs in fish, play a crucial role in several processes, including respiration, osmoregulation, maintaining acid-base balance, and excreting nitrogen waste. Given their direct exposure to the external water environment, the gills are more susceptible to environmental influences compared to other organs [71]. Therefore, by observing the changes in the morphology and structure of the gill tissue, we can judge the impact of the aquaculture environment on the health of the fish [72]. The gill tissues of mandarin fish were structurally intact in this study but showed different degrees of tissue damage, including secondary gill lamella hyperplasia and an increase in the volume of interlamellar cell mass. This may be due to the suspended solids in the BFT aquaculture systems coming into contact with the gill filaments of the mandarin fish through water, and the fish’s body making adaptive changes to protect the gills from excessive suspended solids in the external environment [73]. Compared with the BF group, the secondary gill lamella on both sides of the gill filaments was curved in the RS group and showed hyperplasia. It has been reported that high concentrations of TAN and NO_2_^−^-N in the water environment can cause serious damage to the gill tissue of fish, specifically manifested as pathological phenomena such as epithelial cell shedding, necrosis, proliferation, and fusion of gill lamellae [74]. However, there were seven days of high TAN levels in the RS group, but no enhancement in tissue damage was observed in the gills of mandarin fish. This is likely due to the fact that the BFT aquaculture system can mitigate the degree of gill tissue damage caused by environmental factors. When *Ompok bimaculatus* was subjected to culture water with a TAN concentration of 12.19 mg/L, the BFT group exhibited less gill tissue damage compared to the control group [70].

Proteobacteria, Bacteroidota, Actinobacteriota, and Chloroflexi were the dominant microorganisms in the water in this study, aligning with previous reports that these are the most common bacteria in BFT aquaculture systems [35,75]. Only one species of bacteria in water was identified to the species level, whereas the others were only annotated to the genus or family level. The maintenance of low levels of TAN in the BF group may be attributed to the increased abundance of *Pseudomonas_mosselii*, which is effective in removing ammonia nitrogen, with 98% of ammonium being removed within 24 h when the carbon source is adequate [76]. As indicated by prior research, introducing various carbon sources significantly influences the bacterial communities within BFT aquaculture systems [20,30]. The RS group showed a substantial increase in Alphaproteobacteria (*p* < 0.05) and an increase in *Emticicia* in the experimental water when compared to the BF group.

Research shows that one of the primary sources of bacteria in fish intestinal microbiota in aquaculture systems is the aquatic environment [77,78,79]. In this study, the experimental water showed the highest level of diverse OTUs and bacterial flora, and about half of the intestinal OTUs were found in the water (Figure S1). The findings of this study align with those reported in prior research [80,81]. Fish intestinal microflora play an important role in the health of the fish organism, including nutritional function, immunomodulation, and inhibition of pathogenic bacteria [82]. Proteobacteria overgrowth is considered a potential diagnostic indicator for intenstinal ecological dysbiosis and illness [83]. Proteobacteria dominated the intestinal flora of mandarin fish reared in nets and RAS with a relative abundance of more than 50% [84,85]. In contrast, the BFT aquaculture system revealed that the intestinal tract of mandarin fish contained 31.57 ± 14.41% (RS group) and 14.26 ± 6.51% (BF group) of Proteobacteria. The relative abundance of Fusobacteriota accounted for 31.13 ± 16.44% (RS group) and 30.49 ± 26.61% (BF group) in this study, which was consistent with the fact that Fusobacteriota is highly enriched in the intestinal tract of carnivores and is associated with a protein-rich diet [86]. This indicates that the intestinal health and growth of mandarin fish may benefit from the use of the BFT aquaculture system with rice straw and bamboo flour as carbon sources. *Plesiomonas_shigelloides*, the sole species identified in the RS group’s intestine, is typically observed in both freshwater fish and freshwater ecosystems [86]. Moreover, it is a prominent pathogen, chiefly implicated in intestinal diseases [87]). However, *Plesiomonas_shigelloides* may be considered as a ‘natural vaccine’ against Shigellosis, which gave complete protection against *Shigella sonnei* infection in the animal model [88]. In the intestine of the BF group, *Bacillus_halodurans* and *Caldalkalibacillus_thermarum* types of bacteria were identified. *Caldalkalibacillus_thermarum* is closely related to the Bacillales order, but its function in fish is unclear [89]. *Bacillus_halodurans*, originally isolated from soil, is known for its capacity to produce a two-peptide lantibiotic known as haloduracin [90,91].

## 5. Conclusions

In conclusion, BFT aquaculture systems provide a high-quality water environment that is crucial to the overall health and survival of farmed animals. An essential component of farmed fish’s growth and development is their daily feed intake, which is influenced by the amount of TAN and NO_2_^−^-N in the water. This underscores the importance of maintaining high-quality water in aquaculture systems. Interestingly, despite the BF group exhibiting lower nitrogenous levels and the expression levels of nitrogen functional genes being higher in water compared to the RS group, both groups demonstrated similar levels of microorganism diversity in water and growth performance of mandarin fish. Additionally, mandarin fish showed different degrees of gill tissue damage. Therefore, further research is merited into the association between organic carbon and fish health in BFT systems. Gaining an understanding of this relationship is directly related to optimizing fish production in BFT systems.

## Figures and Tables

**Table 1:** Growth performance of mandarin fish cultured in the RS and BF groups.

	RS	BF	*p*-Value
IBW	38.23 ± 0.17	38.80 ± 0.18	0.082
FBW	81.14 ± 0.88	78.45 ± 2.58	0.380
SR	100.00 ± 0.00	93.33 ± 3.85	0.158
FI	1.56 ± 0.02	1.76 ± 0.11	0.207
WG	112.21 ± 1.56	100.92 ± 6.45	0.164
FCR	1.13 ± 0.02	1.40 ± 0.15	0.153
SGR	1.34 ± 0.01	1.24 ± 0.06	0.166

Results are presented as the means ± SEM (*n* = 3); IBW, initial mean body weight (g fish^−1^); FBW, final mean body weight (g fish^−1^); SR, survival rate (%); WG, weight gain (%); FI, feed intake (% day^−1^). FCR, feed conversion rate. SGR, specific growth ratio (% day^−1^).

**Table 2:** The OTUs and alpha diversity indexes (Chao1 and Shannon) of both water and intestinal samples.

	RS	BF	*p*-Value
Water			
OTUs	732.00 ± 58.28	748.67 ± 110.64	0.900
Chao 1	886.75 ± 61.25	949.71 ± 100.57	0.621
Shannon	4.24 ± 0.37	3.48 ± 0.75	0.417
Intestine			
OTUs	271.33 ± 108.89	369.00 ± 88.09	0.524
Chao 1	327.49 ± 103.92	488.28 ± 96.76	0.321
Shannon	2.21 ± 0.73	2.50 ± 0.82	0.806

Results are presented as the means ± SEM (*n* = 3).

## Data Availability

The original contributions presented in this study are included in the article and Supplementary Materials. Further inquiries can be directed to the corresponding author.

## Supplementary Materials

The following supporting information can be downloaded at: https://www.mdpi.com/article/10.3390/microorganisms13071631/s1, Table S1: Primers used for qPCR [39,92,93,94]; Figure S1: The Venn diagram is used to compare the OTUs of the bacterial community in the water and intestine of the RS and BF groups; Table S2: The relactive abundance of bacteria (%).
