# Supplementary material for: Evaluation of the Application Effects of Siniperca chuatsi in Biofloc Systems: A Comparative Study on the Use of Bamboo Flour and Rice Straw as Carbon Sources
Source: Microorganisms. 2025 Jul 10;13(7):1631. doi: 10.3390/microorganisms13071631 (PMC12299561; doi:10.3390/microorganisms13071631)
Supplement: Supplementary file 1 [file microorganisms-13-01631-s001.zip › microorganisms-3693229-supplementary.pdf]

microorganisms

Table S1. Primers used for qPCR.

Target gene	E (%)	Sequence (5'-3')
16s rDNA[93]	104	ACTCCTACGGGAGGCAGCAG<br>GGACTACHVGGGTWTCTAAT
Anammox[94]	92	GCCGTAAACGATGGGCACT<br>AACGTCTCACGACACGAGCTG
narG[39]	99	CCGATYCCGGCVATGTCSAT<br>GGNACGTTNGADCCCCA
napA[39]	91	GCNCCNTGYMGNTTYTGYGG<br>DATNGGRTGCATYTCNGCCATRT
nirS[39]	100	GTSAACGTSAAGGA R ACSGG<br>GASTTCGGRTGSGTCTTGA
nirK[39]	100	GGMATGGTKCCSTGGCA<br>GCCTCGATCAGRTTRTGGTT
Nosz[95]	106	CGCRACGGCAASAAGGTSMSSGT<br>CAKRTGCAKSGCRTGGCAGAA

E is the amplification efficiency.

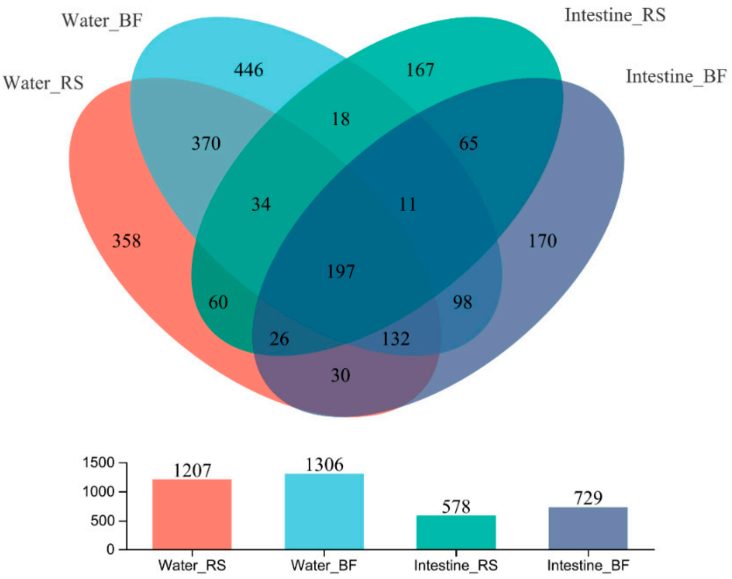

Figure S1. The Venn diagram is used to compare the OTUs of the bacterial community in the water and intestine of the RS and BF groups.

Table S2. The relactive abundance of bacteria (%).

		**water**		**intestine**	
	Taxonomy	RS	BF	RS	BF
Phylum	Fusobacteriota	0.00±0.00	0.00±0.00	31.13±16.44	30.49±26.61
	Proteobacteria	43.58±5.48	26.74±7.82	31.57±14.41	14.26±6.51
	Bacteroidota	19.78±14.88	33.3±14.87	0.00±0.00	0.00±0.00
	Actinobacteriota	13.05±2.37	21.78±4.15	14.31±7.84	30.72±14.25
	Firmicutes	0.00±0.00	0.00±0.00	15.48±11.80	17.23±16.46

	Chloroflexi	15.92±4.90	9.13±7.12	3.11±1.67	4.80±3.25
Class	Fusobacteriia	0.00±0.00	0.00±0.00	31.13±16.44	30.49±26.61
	Bacteroidia	19.77±14.88	33.30±14.87	0.00±0.00	0.00±0.00
	Alphaproteobacteria	31.36±6.22[a]	10.49±1.22[b]	14.49±11.06	7.89±5.59
	Actinobacteria	8.88±1.48	20.26±4.57	13.24±7.17	28.71±12.81
	Gammaproteobacteria	12.19±2.27	16.25±6.85	17.08±10.18	6.37±1.48
	Clostridia	0.00±0.00	0.00±0.00	14.81±12.07	7.04±6.88
	Bacilli	0.00±0.00	0.00±0.00	0.53±0.26	10.10±9.49
	Anaerolineae	6.98±4.40	6.61±5.43	0.00±0.00	0.00±0.00
	Chloroflexia	8.05±4.23	2.23±1.48	2.78±1.57	3.22±2.09
	Acidimicrobiia	2.88±1.15	0.96±0.35	0.00±0.00	0.00±0.00
Order	Chitinophagales	1.67±0.27	26.90±18.00	0.00±0.00	0.00±0.00
	Fusobacteriales	0.00±0.00	0.00±0.00	30.49±26.61	31.13±16.44
	Cytophagales	13.40±12.17	3.60±3.18	0.00±0.00	0.00±0.00
	Corynebacteriales	4.16±1.96	7.90±3.37	21.84±10.96	11.80±6.78
	Peptostreptococcales-Tissierellales	0.00±0.00	0.00±0.00	6.95±6.84	14.4±12.24
	Rhizobiales	13.84±2.35	5.97±0.06	4.68±3.54	12.8±10.25
	Burkholderiales	4.99±1.47	6.32±1.69	1.51±0.10	7.20±3.78
	Enterobacterales	0.00±0.00	0.00±0.00	0.02±0.01	8.55±8.45
	Bacillales	0.00±0.00	0.00±0.00	7.04±6.61	0.19±0.10
	PeM15	0.29±0.16	10.23±4.45	5.15±2.91	0.11±0.08
	Thermomicrobiales	8.04±4.23	2.16±1.42	3.21±2.09	2.77±1.56
	Rhodobacterales	7.81±3.80	1.68±0.75	1.96±1.70	1.05±0.39
	Caldalkalibacillales	0.00±0.00	0.00±0.00	2.79±2.78	0.00±0.00
	Pseudomonadales	1.96±1.43	5.03±3.68	2.14±1.56	0.45±0.20
	Flavobacteriales	4.07±3.21	2.45±1.75	0.00±0.00	0.00±0.00
	Caldilineales	0.69±0.30	5.13±4.78	0.00±0.00	0.00±0.00
	Caulobacterales	4.75±4.14	0.38±0.17	0.00±0.00	0.00±0.00
	RBG-13-54-9	4.08±4.03	0.21±0.08	0.00±0.00	0.00±0.00
Family	Chitinophagaceae	1.13±0.41	24.97±18.71	0.00±0.00	0.00±0.00
	Spirosomaceae	12.81±12.41	3.32±3.25	0.00±0.00	0.00±0.00
	Fusobacteriaceae	0.00±0.00	0.00±0.00	31.13±16.44	30.49±26.61
	Mycobacteriaceae	3.84±1.86	7.79±3.33	11.44±6.63	21.73±10.95
	Peptostreptococcaceae	0.00±0.00	0.00±0.00	14.24±12.32	6.91±6.83
	Rhizobiales_Incertae_Sedis	6.00±2.25[a]	2.75±1.14	8.20±7.86	0.92±0.62
	JG30-KF-CM45	8.02±4.23	2.02±1.30	2.77±1.56	3.19±2.08
	Rhodobacteraceae	7.81±3.80	1.68±0.75	1.05±0.39	1.96±1.70
	Caldilineaceae	0.69±0.30	5.13±4.78	0.00±0.00	0.00±0.00
	Caulobacteraceae	4.52±4.06	0.07±0.04	0.00±0.00	0.00±0.00
	Flavobacteriaceae	3.70±3.28	0.68±0.32	0.00±0.00	0.00±0.00
	Pseudomonadaceae	0.30±0.11	3.14±2.30	0.00±0.00	0.00±0.00
	Saprospiraceae	0.30±0.24	1.84±1.69	0.00±0.00	0.00±0.00
	Enterobacteriaceae	0.00±0.00	0.00±0.00	8.54±8.45	0.01±0.01
	Alcaligenaceae	0.00±0.00	0.00±0.00	6.84±3.54	1.20±0.22
	Bacillaceae	0.00±0.00	0.00±0.00	0.18±0.10	7.03±6.60
	Caldalkalibacillaceae	0.00±0.00	0.00±0.00	0.00±0.00	2.79±2.78
Genus	Terrimonas	0.34±0.10	24.38±18.91	0.00±0.00	0.00±0.00
	Emticicia	12.54±12.54	0.01±0.01	0.00±0.00	0.00±0.00
	Cetobacterium	0.00±0.00	0.00±0.00	31.13±16.44	30.49±26.61
	Mycobacterium	3.84±1.86	7.79±3.33	11.44±6.63	21.73±10.95
	Gemmobacter	4.08±2.50	0.67±0.31	0.00±0.00	0.00±0.00
	Flavobacterium	3.69±3.28	0.66±0.31	0.00±0.00	0.00±0.00
	Pseudomonas	0.30±0.11	3.14±2.30	0.00±0.00	0.00±0.00

	Plesiomonas	0.00±0.00	0.00±0.00	8.51±8.47	0.00±0.00
	Achromobacter	0.00±0.00	0.00±0.00	6.84±3.54	1.20±0.22
	Bacillus	0.00±0.00	0.00±0.00	0.15±0.07	6.49±6.07
	Caldalkalibacillus	0.00±0.00	0.00±0.00	0.00±0.00	2.79±2.78
Species	Pseudomonas_mosselii	0.24±0.11	3.04±2.28	0.00±0.00	0.00±0.00
	Plesiomonas_shigelloides	0.00±0.00	0.00±0.00	8.51±8.47	0.00±0.00
	Bacillus_halodurans	0.00±0.00	0.00±0.00	0.00±0.00	3.91±3.91
	Caldalkalibacillus_thermarum	0.00±0.00	0.00±0.00	0.00±0.00	2.79±2.78

Results are presented as the means ± SEM (n = 3). Different lowercase letters indicate significant differences between treatments $(P < 0.05)$.
